# The Gut Microbial Metabolite Trimethylamine-*N*-Oxide Is Present in Human Cerebrospinal Fluid

**DOI:** 10.3390/nu9101053

**Published:** 2017-09-22

**Authors:** Daniele Del Rio, Francesca Zimetti, Paolo Caffarra, Michele Tassotti, Franco Bernini, Furio Brighenti, Andrea Zini, Ilaria Zanotti

**Affiliations:** 1 Dipartimento di Scienze degli Alimenti e del Farmaco, Università degli Studi di Parma, Parco Area delle Scienze 27/A, 43124 Parma, Italy; daniele.delrio@unipr.it (D.D.R.); francesca.zimetti@unipr.it (F.Z.); michele.tassotti@studenti.unipr.it (M.T.); f.bernini@unipr.it (Fr.B.); furio.brighenti@unipr.it (Fu.B.); 2 Dipartimento di Medicina e Chirurgia, Unità di Neuroscienze, Università degli Studi di Parma, via Gramsci 14, 43126 Parma, Italy; paolo.caffarra@unipr.it; 3 Dipartimento di Neuroscienze, Nuovo Ospedale Civile “S.Agostino-Estense”, Azienda Ospedaliera Universitaria, via Giardini 1355, 41100 Modena, Italy; andrea.zini@me.com

**Keywords:** trimethylamine-*N*-oxide, gut microbiota, central nervous system

## Abstract

Trimethylamine-*N*-oxide (TMAO) is a small organic molecule, derived from the intestinal and hepatic metabolism of dietary choline and carnitine. Although the involvement of TMAO in the framework of many chronic diseases has been recently described, no evidence on its putative role in the central nervous system has been provided. The aim of this study was to evaluate whether TMAO is present at detectable levels in human cerebrospinal fluid (CSF). CSF was collected for diagnostic purposes from 58 subjects by lumbar puncture and TMAO was quantified by using liquid chromatography coupled with multiple-reaction monitoring mass spectrometry. The molecule was detected in all samples, at concentrations ranging between 0.11 and 6.43 µmol/L. Further analysis on CSF revealed that a total of 22 subjects were affected by Alzheimer’s disease (AD), 16 were affected by non-AD related dementia, and 20 were affected by other neurological disorders. However, the stratification of TMAO levels according to the neurological diagnoses revealed no differences among the three groups. In conclusion, we provide the first evidence that TMAO can be assessed in human CSF, but the actual impact of this dietary metabolite in the patho-physiolgy of the central nervous system requires further study.

## 1. Introduction

It is now generally accepted that gut microbiota represents a major regulator of health and disease in humans, both contributing to the host metabolic and immune functions and potentially driving the onset of chronic diseases [1]. Among the mechanisms accounting for the pathological implications of the microbiota, the metabolism of dietary-derived products is currently a matter of wide interest. As an example, the ingestion of food rich in choline (dairy products, eggs, fish) or carnitine (red meat) is associated with increased plasma levels of trimethylamine-*N*-oxide (TMAO) through the sequential action of intestinal microbes and the hepatic flavin monooxigenase 3 (FMO3) [2]. This small organic molecule has drawn the attention of the scientific world following the demonstration that high plasma levels are associated with an increased risk of cardiovascular events [3,4,5]. Whereas the existence of a reciprocal influence between gut and brain is not a matter of debate, no evidence of clinical implications of TMAO in the central nervous system has yet been documented. In order to address this issue, we assessed TMAO in cerebrospinal fluid (CSF) collected from 58 subjects that underwent a lumbar puncture for diagnostic purposes and demonstrate for the first time that TMAO is present at detectable levels in the central nervous system.

## 2. Materials and Methods

CSF were obtained at the Neurology Unit of the University of Parma for diagnostic purposes after informed consent. The study was approved by The Institutional Review Board of the University of Parma (authorization number 0058/2017). Samples were collected in the morning, after one night of fasting, and immediately stored at −80 °C.

For TMAO quantification, before the analysis, each sample was added with TMAO-d9 as internal standard and then extracted with acidified acetonitrile, as previously described [6]. Samples were centrifuged and the supernatants collected and analyzed by a UHPLC DIONEX Ultimate 3000 equipped with a triple quadrupole TSQ Vantage (Thermo Fisher Scientific Inc., San Josè, CA, USA) fitted with a heated-ESI (H-ESI) (Thermo Fisher Scientific Inc., San Jose, CA, USA) probe. Separations were carried out by means of an XBridge BEH HILIC XP (100 mm × 2.1 mm) column, with a 2.5 μm particle size (Waters, Milford, MA, USA). Statistical analyses were performed using Prism 6.0 (GraphPad Inc., San Diego, CA, USA).

Alzheimer’s Disease (AD) (*n* = 22) and non-AD related dementia (non-AD) (*n* = 16) was diagnosed according to International Working Group (IWG)-2 criteria [7]. Non-AD includes frontotemporal dementia, corticobasal degeneration, and degenerative Parkinsonism. Aβ 1-42, tau, and phospho-tau levels were evaluated via enzyme-linked immunosorbent assay (ELISA) (Fujirebio, Ghent, Belgium). The third group included age- and sex-matched subjects that experienced neurological disorders unrelated to demyelinating inflammatory disorders, stroke, and neurodegenerative and infective diseases.

## 3. Results

**Figure 1:**
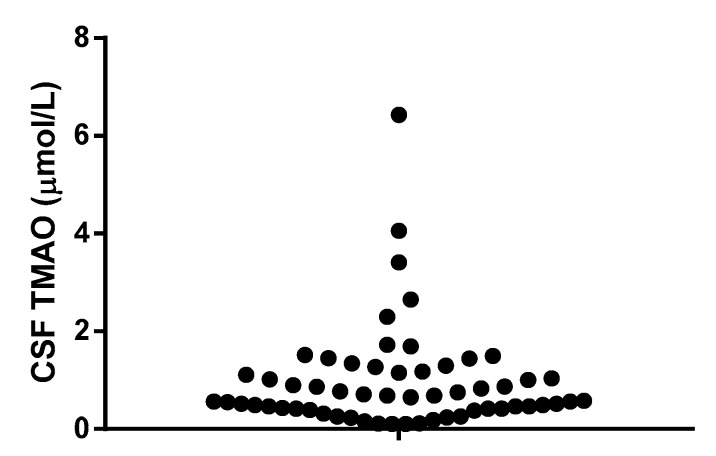
Trimethylamine-*N*-oxide (TMAO) concentrations in human cerebrospinal fluid (CSF). TMAO was quantified as described in the method section.

All tested samples showed detectable amounts of TMAO, in a range between 0.11 and 6.43 µmol/L (median: 0.665 µmol/L, 95% CI-0.490 to 0.870 µmol/L) (Figure 1).

Successively, the data were stratified according to the neurological diagnoses following the analysis of CSF. Demographic data and dementia diagnostic parameters of subjects are shown in Table 1.

As expected, patients with prodromal AD had significantly decreased Aβ 1-42 and increased tau and phospho-tau in CSF compared to non-AD subjects.

No differences in the actual concentrations of TMAO were observed among the three groups (one-way ANOVA *p* = 0.326).

## 4. Discussion

TMAO has been recently proposed as a novel prognostic marker of cardiovascular events, given the association of its plasma levels with indexes of atherosclerotic plaque vulnerability [8], major cardiac events [5], and death [9]. Similarly, it has been associated with other chronic diseases, such as diabetes [10] and cancer [11], further strengthening the pathological implications of this microbiota metabolite.

The recognized connections between gut and brain has recently piqued interest in the impact of diet and microbiota on the functionality of the central nervous system. Up to now, only one work on lactic acid and short chain fatty acids have revealed how dietary-derived products may exert (potentially adverse) neurological effects [12].

The present study originates from the hypothesis that TMAO may play a direct role in the central nervous system. As a preliminary, fundamental aspect, its presence in the CSF needed to be assessed, and this was the main aim of this short study. A recent work, based on an innovative microphysiology system hypothesized that TMAO could cross the blood–brain barrier [13], but this has never been demonstrated in vivo. For the first time, TMAO has been measured in human CSF, reaching quantifiable levels in all tested samples. Unfortunately, plasma samples from the same subjects were not available, preventing any correlation analysis between biological fluids. Published data from large epidemiological studies revealed that TMAO in plasma may fluctuate in a very wide range of concentrations (0.08–250 µmol/L) and that it is affected by several factors, including dietary intake of choline and carnitine-containing food, the composition of microbiota, and the activity of FMO3 [2]. The levels we detected are consistent with the hypothesis that a small fraction of liver-derived TMAO can cross the blood–brain barrier, but we cannot rule out that a fraction of the TMAO detected in CSF may derive from de novo synthesis, as expression of FMO3 has been detected in the adult brain [14].

In the small tested group of subjects, TMAO levels in CSF are apparently unrelated to the diagnosed neurological disorders. However, establishing the prognostic value of TMAO is beyond the scope of this study, so it is not yet possible to draw conclusions on this aspect.

Albeit preliminary, this study introduces an interesting scenario about the actual role of TMAO in modulating functions of the central nervous system. Future studies in proper in vitro and in vivo models will address this issue, adding novel pieces to the gut–brain axis puzzle.

## Figures and Tables

**Table 1:** Demographic data and dementia diagnostic parameters.

	AD (*n* = 22)	Non-AD (*n* = 16)	Other Neurological Disorders (*n* = 20)
Age (years)	69 ± 9	63 ± 8	65 ± 17
Male sex, *n* (%)	12 (54)	9 (56)	10 (50)
Aβ 1–42 (ng/L)	416 (177–674)	675 *** (288–1540)	N.E.
Tau (ng/L)	449 (109–1644)	204 *** (22–564)	N.E.
Phospo-Tau (ng/L)	80 (25–742)	33 *** (3–64)	N.E.
TMAO (µmol/L)	0.520 (0.32–1.15)	0.710, (0.56–1.50)	0.570, (0.42–1.00)

Data are expressed as mean ± S.D. for normally distributed values or as median (interquartile range) for data that are not normally distributed. The one way ANOVA test was applied to compare the three groups for age; the non-parametric two-tailed Mann Whitney test was applied to compare AD and non-AD subjects for CSF neurobiomarkers. *** *p* < 0.01 vs. AD. AD: Alzheimer’s disease; non-AD: non-AD related dementia. N.E.: not evaluated.
